# Prevalence and correlates of multimorbidity among patients attending AYUSH primary care settings in Delhi-National Capital Region, India

**DOI:** 10.1186/s12906-023-04158-7

**Published:** 2023-11-29

**Authors:** Roja Varanasi, Abhinav Sinha, Debadatta Nayak, Raj K. Manchanda, Rajiv Janardhanan, Simran Tandon, Sanghamitra Pati

**Affiliations:** 1 Amity Institute of Public Health, Amity University, Noida, India; 2 Central Council for Research in Homoeopathy, Ministry of AYUSH, New Delhi, India; 3 ICMR-Regional Medical Research Centre, Bhubaneswar, Odisha India; 4 Directorate of AYUSH, Government of Delhi, Delhi, India; 5 SRM Institute of Science & Technology, Chennai, Tamil Nadu India; 6 Amity School of Health Sciences, Amity University, Mohali, Punjab India

**Keywords:** Multimorbidity, Primary care, AYUSH, Delhi, India

## Abstract

**Introduction:**

India has a multifaceted healthcare system and recognizes complementary and alternative systems of medicine (AYUSH) that cater to the healthcare needs of people. Multimorbidity requires frequent visits to physicians and long-term use of medications, due to which people tend to prefer AYUSH systems as they provide holistic patient-centered treatment. Hence, we aimed to estimate the prevalence of multimorbidity and assess its correlates among patients attending AYUSH primary care clinics in Delhi.

**Methods:**

A cross-sectional study was conducted among 943 patients aged ≥ 18 years attending various AYUSH primary care clinics in Delhi from September 2021 to February 2022, employing a stratified random sampling technique. Descriptive statistics such as frequency and proportion were used to report the prevalence of multimorbidity (two or more chronic conditions in an individual out of the 33 conditions listed as per the Multimorbidity Assessment Questionnaire for Primary Care). A multivariable logistic regression assessed the association between various socio-demographic characteristics and multimorbidity, presented as an adjusted odds ratio (AOR) with a 95% confidence interval (CI).

**Results:**

The prevalence of diabetes (14.7%) was found to be the highest (out of all included chronic conditions) among the patients attending various AYUSH primary care settings. The overall prevalence of multimorbidity was observed to be around 39.4%. We observed a higher likelihood of having multimorbidity among participants aged ≥ 70 years [AOR: 9.19 (95% CI: 3.75–22.54)], females [AOR: 1.57 (95% CI: 1.04–2.37)], and middle class [AOR: 2.23 (95% CI: 1.45–3.43)].

**Conclusion:**

Multimorbidity was evidently prevalent across AYUSH primary care settings, which cannot be overlooked. The results suggest behavioral change communication may be aimed at older individuals, females, and the middle class.

**Supplementary Information:**

The online version contains supplementary material available at 10.1186/s12906-023-04158-7.

## Introduction

The simultaneous presence of two or more chronic conditions in an individual, known as multimorbidity, is evidently rising in low- and middle-income countries (LMICs) [1]. A systematic review observed the pooled prevalence of multimorbidity to be around 36.4% in LMICs [2]. Prolonged longevity along with changing lifestyles have led to a rise in multimorbidity in LMICs such as India, where chronic conditions usually present a decade earlier than in high-income countries [3]. This could be due to compounding factors such as poverty, limited healthcare infrastructure, and deficient coping mechanisms [4]. Multimorbidity is associated with higher healthcare utilization and out-of-pocket expenditure [5]. It is also linked with poorer patient-reported outcome measures such as health-related quality of life, physical functioning, falls, and psychological distress [6, 7]. Intake of multiple drugs (polypharmacy) among multimorbid individuals may also lead to a greater economic burden [8]. Multimorbid individuals are at a higher risk of seeking emergency care, inpatient services, and may die prematurely [9]. Evidence suggests age is one of the strongest predictors of multimorbidity [10]. However, in absolute terms, more young adults and those in midlife have multimorbidity [10, 11], which could partly be attributed to the higher proportion of these individuals in the general population, especially in India.

The concept of multimorbidity advocates for patient-centered care over the prioritization of single conditions [12]. Although existing clinical guidelines focus on a single chronic condition, they often conflict with clinical decision-making. Nonetheless, primary care remains the first and foremost point of contact for the majority of multimorbid individuals [6]. The findings of a systematic review suggest that the prevalence of multimorbidity in primary care ranged from 12.9% to 95.1% [13]. Indian healthcare system comprises both government-funded public and self-financed private sectors. Amongt the public, there exists a three-tier structure with primary, secondary, and tertiary specialized clinics. Further, the primary care facilities support conventional allopathic treatment along with the recent incorporation of indigenous complementary and alternative systems of medicine popularly termed AYUSH (Ayurveda, Yoga and Naturopathy, Unani, Siddha, Sowa Rigpa and Homeopathy). The AYUSH system of medicine is highly trusted for long-term conditions as it manages patients with a holistic approach [14, 15]. Additionally, management of chronic conditions requires long-term use of medications that might have side effects in the allopathic systems, due to which patients resort to AYUSH systems [16]. Moreover, evidence suggests that AYUSH treatment through integrative care for chronic conditions has been successful [17–19].

Although the burden of multimorbidity in allopathic primary care settings has been estimated across various studies in India [20–22], but, there is a dearth of evidence on the burden of multimorbidity among patients attending AYUSH primary care settings. The recent Health and Wellness Centres established to strengthen primary care also include an AYUSH dispensary. This study will generate evidence on the burden of multimorbid patients attending these clinics that will be helpful in deciding future programs and policies around multimorbidity in AYUSH care settings. We aimed to estimate the prevalence and assess the correlates of multimorbidity among adult patients attending AYUSH primary care clinics in the National Capital Region of Delhi, India.

## Methods

### Study design and setting

A cross-sectional study was conducted among various AYUSH primary healthcare dispensaries in the Delhi National Capital Territory (NCT) from September 2021 to February 2022. Delhi is a megacity located in North India where people from various parts of India migrate for education and livelihood. We obtained information on AYUSH primary care settings from the Directorate of AYUSH, Delhi. There are 106 Homeopathic dispensaries, 40 Ayurvedic clinics, and 20 Unani dispensaries in Delhi NCT.

### Study participants

We included adult patients (18 years and older) attending the out-patient departments of AYUSH primary care clinics. Patients too ill to participate and those with insufficient cognitive ability to complete the questionnaire were excluded from the study. To avoid duplication, the participants were interviewed only once at a facility, and they were excluded if they were known to have been interviewed earlier at any other facility.

### Sample size

Based on a previous study from India, we considered the prevalence of multimorbidity to be around 23% among patients attending primary care settings [20], with 15% relative precision, a 95% confidence interval (CI), and a design effect of 1.5, a minimal sample of 833 was required. After accounting for the 15% non-response rate, the final required sample size was 958. However, only 943 participants were enrolled.

### Sampling

A list of all AYUSH dispensaries under the Directorate of AYUSH, NCT, Delhi, was obtained, from which we randomly enrolled 20% of dispensaries of each AYUSH system following the proportional probability sampling technique thus covering 20 Homeopathy clinics, eight Ayurveda clinics, and four Unani clinics. Thereafter, from each dispensary, 30 random individuals were enrolled following systematic random sampling. We enrolled every fifth individual from each dispensary to complete the total sample of 943.

### Data collection and management

Data was collected by AYUSH clinicians using the Multimorbidity Assessment Questionnaire for Primary Care (MAQ-PC), a previously validated tool that was developed through an iterative process and adapted for the present study at AYUSH primary care settings (Supplementary File S1) [23]. Based on MAQ-PC questionnaire and thorough literature review, we included 33 chronic conditions as follows: arthritis, diabetes, hypertension, chronic lung diseases, acid peptic disease, chronic backache, chronic heart diseases, stroke, visual impairment, hearing impairment, dementia, alcohol disorder, cancer, chronic kidney disease, epilepsy, thyroid disorders, irritable bowel syndrome, chronic constipation, prostate condition, sleep disorders, chronic liver diseases, psoriasis, eczema, chronic rhinosinusitis, chronic headache, depression, vitiligo, anxiety, chronic cervical/neck pain, polycystic ovarian disease/syndrome, fungal infection, uterine fibroid/tumor, vaginal discharge/leucorrhoea/cervicitis. All conditions considered were self-reported in nature, though they were triangulated with the prescriptions of the patients by the data collector (clinician). Socioeconomic class was based on the Modified Kuppuswamy socioeconomic scale based on education of the head of household (score: 10 to 1), occupation of the head of family (score: 7 to 1), and total monthly income of the family (score: 12 to 1), updated for the year 2021 [24]. The socio-economic classes were grouped as ‘upper’ (maximum score: 29–26) ‘middle’ (score: 25–15) and ‘lower’ (score: 10- < 5) based on the scores obtained from the modified Kuppuswamy scale. Education was categorized as ‘no formal education’: those with no education; ‘primary’: those with five years of schooling; and ‘secondary and above’: those with six years or more of schooling. Data on socio-demographic characteristics and self-reported chronic conditions was collected to assess multimorbidity using Open Data Kit (ODK), an Android-based software. We grouped ethnicity as ‘aboriginal’: those who reported themselves as tribal, and all others as ‘non-aboriginal’. All AYUSH clinicians selected for collecting data were provided withcomprehensive training to standardize the data collection procedures. The investigators trained the clinicians following a pre-laid standard protocol to maintain consistency. The investigators personally monitored the data collection to review the adherence to protocol. Additionally, 10% of the data was randomly checked to ensure accuracy.

### Statistical analysis

Data was downloaded from the server in a Microsoft Excel spreadsheet. The data were thoroughly checked for missing values, duplicates, and outliers. Continuous variables such as age were presented as the mean and standard deviation. Descriptive statistics such as frequency and proportion were used to report the prevalence of each chronic condition. Multimorbidity (the main outcome of interest) was assessed using a simple count of the number of self-reported chronic conditions present, with two or more chronic conditions termed multimorbidity. A chi-square test was used to assess the significance, and a *p*-value of < 0.05 was considered significant. For supplementary Table S2, we used Yate’s correction to assess the significance wherever the prevalence of chronic conditions was zero. Bivariate logistic regression assessed the association between individual socio-demographic characteristics and multimorbidity, presented as an odds ratio (OR) with 95% CI. A multivariable logistic regression model presented as an adjusted odds ratio (AOR) with 95% CI was used to assess the adjusted association between socio-demographic variables and multimorbidity. We used SPSS v25.0 (SPSS IBM) to analyze the data.

### Ethical considerations

This study was approved by the Institutional Ethics Committee of Amity University, Noida (vide AUUP/IEC/2021-JAN/04) dated 12^th^ March, 2021. Additionally, we also obtained permission from the Directorate of AYUSH, Govt. of NCT Delhi. Informed written consent was obtained from the study participants prior to enrollment. The confidentiality of the participants was maintained as anonymous data was stored with minimal access.

## Results

We approached 1256 patients, out of whom 943 agreed to participate in the study (30% non-response rate). The mean age of the participants was observed to be 42.07 ± 14.05 years. Around one-fourth of the participants belonged to the 40–49 age group. We observed that 53% of the respondents were female, while 19% of the participants belonged to the aboriginal group. Almost equal proportions of respondents were employed and unemployed. Three-fourths of the respondents were married. A majority of the participants (56%) belonged to lower socioeconomic classes. The detailed socio-demographic profile of patients visiting various AYUSH clinics is presented in Table 1.

**Table 1:** Socio-demographic characteristics of study population across various AYUSH clinics

Characteristics	Total (*N* = 943)	Ayurveda (*N* = 246)	Unani (*N* = 121)	Homoeopathy (*N* = 576)	*p*-value
	n (%)	n (%)	n (%)	n (%)	
**Age (Years)**	42.07 ± 14.05	^a^43.5 ± 13.2	^a^42.4 ± 15.4	^a^41.4 ± 14.1	0.12
18–29	192(20.4)	39 (15.9)	24(19.8)	129(22.4)	0.11
30–39	233(24.7)	51(20.7)	33(27.3)	149 (25.9)	
40–49	238(25.2)	77(31.3)	25(20.7)	136(23.6)	
50–59	157(16.6)	46(18.7)	20(16.5)	91(15.8)	
60–69	77(8.2)	24(9.8)	10(8.3)	43(7.5)	
≥ 70	46(4.9)	9(3.7)	9(7.4)	28 (4.9)	
**Sex**					
Male	444(47)	139(56)	60(49.6)	245(42.5)	**0.001**
Female	499 (53)	107(44)	61(50.4)	331(57.5)	
**Education**					
No Formal Education	35(3.5)	14(5.7)	4 (3.3)	7(3)	**0.004**
Primary	203 (21.5)	53(21.5)	40(33.1)	110(19)	
Secondary and above	705(44.8)	179(72.7)	77(63.6)	449(75.9)	
**Occupation**					
Unemployed	416(44.1)	93(37.8)	59(48.8)	264(45.8)	**0.05**
Employed	524(44.3)	151(61.3)	62(51.2)	311(53.9)	
**Marital status**					
Single	233(24.7)	62(25.2)	32(26.4)	139(24.1)	0.84
Married	710(75.3)	184(74.8)	89(73.6)	437(75.9)	
**Ethnicity**					
Aboriginal	131(19)	31(17.1)	9(9.5)	91(22.1)	**0.01**
Non aboriginal	557(81)	150(82.9)	86(90.5)	321(77.9)	
**Socio-economic Class**					
Upper	44(4.7)	9(3.7)	3(52.5)	32(5.6)	**0.0001**
Middle	371(39.3)	96(39.0)	28(23.1)	247(42.9)	
Lower	528(56.0)	141(57.3)	90(74.4)	247(51.6)	
**Morbidity**					
No/Single morbidity	571(60.6)	140(56.9)	63(56.2)	363(63.0)	0.15
Multimorbidity (2 or more chronic conditions)	372(39.4)	106(43.1)	53(43.8)	213(37.0)	

^a^Data are presented in mean ± SD; *AYUSH* Ayurveda, Yoga and Naturopathy, Unani, Siddha and Homeopathy

The prevalence of cardio-metabolic diseases (25.8%) was found to be highest [diabetes (14.7%), hypertension (14.3%)], followed by skin diseases (13.1%), among the patients attending various AYUSH primary care settings. The highest prevalence of cardio-metabolic diseases was also observed among the patients attending Ayurveda clinics, while skin diseases were more prevalent among the respondents visiting Homeopathy clinics (Supplementary Table S2). The overall prevalence of multimorbidity was observed to be around 39.4%. We observed the prevalence of multimorbidity to be 43.1% in Ayurveda clinics, 43.8% in Unani clinics, and 37% in homeopathy facilities (Table 1).

The highest prevalence of multimorbidity (48.3%) was observed among the participants aged 40–49 years. We observed a higher prevalence of multimorbidity among females (41.7%) than their male counterparts. Participants with no formal education had a higher prevalence of multimorbidity. Unemployed respondents had a higher prevalence (42.1%) of multimorbidity (Table 2). Non-aboriginal participants were found to have a higher prevalence (42.5%) of multimorbidity. Considering the socioeconomic groups, the middle class had a higher prevalence (41.5%) of multimorbidity.

**Table 2:** Association of multimorbidity with various socio-demographic characteristics among study population

Characteristics	No/ Single morbidity (*N* = 571)	Multimorbidity (*N* = 372)	Odds Ratio	*p*-value	Adjusted Odds Ratio	*p*-value
**Age (Years)**	n (%)	n (%)				
18–29	151(78.6)	41(21.4)	Ref		Ref	
30–39	158(67.8)	75(32.2)	1.74(1.12—2.71)	**0.013**	2.29(1.30—4.03)	**0.004**
40–49	123(51.7)	115(48.3)	3.44(2.24—5.28)		4.75(2.72—8.31)	**0.0001**
50–59	84(53.5)	73(46.5)	3.20(2.00—5.10)		4.85(2.61—9.02)	**0.0001**
60–69	38(49.4)	39(50.6)	3.78(2.14—6.64)		5.83(2.72—12.49)	**0.0001**
≥ 70	17(37)	29(63)	6.28(3.14—12.53)		9.19(3.75—22.54)	**0.0001**
**Sex**						
Male	280(63.1)	164(36.9)	Ref		Ref	
Female	291(58.3)	208(41.7)	1.22(0.93—1.58)	0.13	1.57(1.04—2.37)	**0.029**
**Education**						
No Formal Education	14(40)	21(60)	Ref		**Ref**	
Primary	112(55.2)	91(44.8)	0.54(0.26—1.12)	0.100	0.62(0.25—1.49)	0.291
Secondary and above	445(63.1)	260(36.9)	0.39(0.19—0.77)	0.008	0.44(0.18—1.04)	0.064
**Occupation**						
Unemployed	241(57.9)	175(42.1)	Ref		Ref	
employed	330(62.6)	197(37.4)	0.82(0.63—1.06)	0.144	0.87(0.55—1.36)	0.544
**Marital status**						
Single	147(63.1)	86(36.9)	Ref		Ref	
Married	424(59.7)	286(40.3)	1.15(0.85—1.56)		0.71(0.46—1.10)	0.132
**Ethnicity**						
Aboriginal	82(62.6)	49(37.4)	Ref		**Ref**	
Non aboriginal	320(57.5)	237(42.5)	1.23(0.83—1.83)	0.283	1.12(0.74—1.70)	0.575
**Socio-economic Class**						
Lower	327(61.9)	201(38.1)	Ref		Ref	
Middle	217(58.5)	154(41.5)	1.02(0.54—1.92)	0.941	2.23(1.45—3.43)	**0.0001**
Upper	27(61.4)	17(38.6)	1.15 (0.88—1.51)	0.299	1.63(0.72—3.70)	0.237

The bivariate regression model identified age as an important predictor of multimorbidity. The chances of having multimorbidity were highest [OR: 6.28 (95% CI: 3.14–12.53)] among the respondents aged ≥ 70 years. The multivariable regression after adjustment showed age, sex, and socio-economic status to be significantly associated with multimorbidity. The participants aged ≥ 70 years had a higher chance [AOR: 9.19 (95% CI: 3.75–22.54)] of having multimorbidity as compared to the youngest age group. Females had higher odds [AOR: 1.57 (95% CI: 1.04–2.37)] of having multimorbidity than their male counterparts. Participants belonging to the middle class had a higher chance [AOR: 2.23 (95% CI: 1.45–3.43)] of having multimorbidity than the most deprived group.

However, we observed multimorbidity to be significantly associated with younger age groups in Ayurveda clinics (Supplementary Table S3). In Homeopathy clinics, multimorbidity was significantly associated with older age groups, females, and the most affluent class (Supplementary Table S4). In Unani clinics, we observed higher odds of multimorbidity among the most affluent patients as compared to the most deprived class (Supplementary Table S5).

## Discussion

Complementary systems of medicine are well-established and trusted for the management of long-term conditions. With a rise in chronic conditions, people have been preferring the AYUSH system to avoid the side effects of long-term allopathic medicines. Hence, we aimed to estimate the prevalence of multimorbidity among patients attending the AYUSH system of medicine. We observed that the prevalence of diabetes was highest, followed by hypertension and skin diseases among the respondents. We found a considerable prevalence of multimorbidity among patients visiting various AYUSH primary care clinics, which further increased with age. Multimorbidity was significantly associated with higher age, females, and the middle class.

We observed that the prevalence of diabetes was highest, followed by hypertension and skin diseases, among the respondents attending AYUSH primary care settings, which is in contrast to the findings of a study conducted among patients attending an Ayurveda clinic in Haryana, India, which reported skin diseases and osteoarthritis to be the most common morbidities [25]. A probable reason for this could be that patients have a higher level of trust in the traditional system of medicine for the management of chronic conditions such as hypertension and diabetes, as these require frequent visits to the physician, consistent investigations, and continued medications for longer durations, which leads to non-adherence. Moreover, AYUSH clinics in Delhi offer free laboratory investigations for all chronic conditions either directly at the clinics or, if the facility is not available at the clinic, the sample is collected at the clinic and tested at an empanelled private laboratory for which the government bears the cost. Health and Wellness Centres also provide free testing for all chronic conditions. Additionally, the fear of side effects from these medications calls patients to shift to other forms of medicine where AYUSH provides a patient-centered holistic management. The other part of the dialogue is that patients with diabetes and hypertension not only suffer from the disease per se but also other symptoms due to additional chronic conditions such as musculoskeletal disease and skin diseases, for which they seek the help of AYUSH systems to avoid polypharmacy and overuse of allopathic medications [26]. Globally, therapeutic dilemmas for physicians prevail in choosing or prioritizing patients’ requests over iatrogenic risks [27].

We observed the prevalence of multimorbidity to be around 39% among patients visiting AYUSH primary care settings, which is higher than the findings of a similar study done among patients attending allopathic primary care facilities in Odisha, India, which observed the burden of multimorbidity to be around 28.3% [28]. Additionally, it was also higher than a study conducted in rural Kerala, India, which reported the prevalence of multimorbidity to be around 16.2% [22]. This highlights that the burden of multimorbid patients is higher in AYUSH primary care as compared to allopathic clinics. Here, it is worth noting that there are limited studies on multimorbidity in AYUSH primary care, which makes comparing our findings with similar studies challenging. Nonetheless, a study conducted among patients utilizing services from complementary and alternative medicine clinics in the USA observed that almost 18% of the participants had two or more chronic conditions [29]. A potential explanation for the higher prevalence observed in our study could be that complementary and alternative medicine is not a mainstay in the USA, whereas most Indians prefer AYUSH for managing chronic conditions.

We found multimorbidity to be significantly associated with higher age, which is consistent with the findings of a systematic review that observed the prevalence of multimorbidity to be higher among older participants [30]. It is also in congruence with the results of a community-based study utilizing nationally representative data from India, which reported multimorbidity to be associated with participants aged 60 years and above [31]. Chronic conditions present around midlife and tend to accumulate thereafter, leading to a rise in their count among older adults. Nonetheless, these older adults may be dependent on their caregivers to access healthcare facilities and providing timely medications. These individuals should be prioritized for providing accessible and affordable primary care at their doorstep which would help in achieving Universal Health Coverage. Here, AYUSH primary care facilities can act as a savior in providing holistic care for these older adults. In the absence of allopathic physicians, especially in hard-to-reach areas, the introduction of AYUSH has helped strengthen primary care, as these are easily accessible and have no user fee.

Our study found females had a higher likelihood of having multimorbidity, which is consistent with the findings of previous studies done among patients visiting primary care in India [28, 32]. In LMICs such as India, women are often vulnerable as they are often economically dependent on other family members. This forces them to step back from prioritizing their own health. Additionally, cultural and societal norms refrain them from making decisions pertaining to their health. Hence, there is a pressing need to address the healthcare needs of women by strengthening AYUSH clinics at all primary care centres. Since these centres would be nearby, they could easily be accessed by the females without having to pay, thereby eliminating dependence.

We observed that participants belonging to the middle class had a higher chance of having multimorbidity, which is dissimilar with most of the existing Indian literature, as these studies report that the most affluent classes has a higher likelihood of having multimorbidity [33, 34]. The literature suggests the affluent class has a higher income and thus can afford diagnosis, due to which they tend to report higher levels of multimorbidity in the self-reported surveys. However, our findings suggest the middle class was more significantly associated with multimorbidity in AYUSH primary care, which reveals that more multimorbid people in this group seek care from these clinics. This is a novel finding that the Indian middle class prefers the AYUSH system for managing of multimorbidity as compared to the richest. It has serious policy implications as these individuals form a high proportion of the population in terms of absolute numbers and, hence, may require more AYUSH clinics in the future.

Implications for policy and practice.

The findings suggest multimorbidity is common in AYUSH primary care settings, which calls for strengthening these clinics as it will facilitate in easy accessibility of services for more patients. Nonetheless, newly proposed Health and Wellness Centres envisage having AYUSH practitioners who can provide holistic care to multimorbid patients. Additionally, interventions such as Yoga and continued support of traditional medicines may help reduce overall out-of-pocket expenditure for patients. Overall well-being can be increased through inter-professional coordination within AYUSH systems, which could be incorporated into the guidelines. Additionally, coordination of AYUSH with other medical/dental professionals (multi-disciplinary teams) for coordinated quality care is also needed [35]. Nonetheless, down referral and up referral to secondary or tertiary levels of care are still important. Family-level approaches to the management of multimorbidity, which include the prevention of shared risk factors, are warranted [36]. Behavioral change communication for prevention is the most important step that AYUSH primary care practitioners should incorporate into their daily clinical practice.

### Strengths and limitations

This is the first ever study in India to estimate the prevalence of multimorbidity across various clinics in AYUSH primary care. We randomly selected clinics as well as patients to increase the generalizability of the study. Although our data was limited by self-reported chronic conditions, it was collected by the clinicians, who also triangulated the self-reported diagnosis with prescriptions. Another limitation of our study was that we excluded patients with cognitive impairment (though most of these patients tend to seek care from AYUSH systems). Although the patients with cognitive impairment were excluded, the numbers were negligible, and that may not have impaired the results much. Our study, being cross-sectional, could not establish causality.

## Conclusion

We observed multimorbidity to be evidently prevalent across AYUSH primary care settings, which cannot be overlooked. The results suggest behavioral change communication to tackle multimorbidity should target higher age individuals, females, and the middle class. Additionally, tailored interventions for managing multimorbidity are required by these groups. Our findings suggest a need for strengthening AYUSH primary care settings in terms of fulfilling the demands of these multimorbid individuals. Future intervention studies among multimorbid patients may be planned at AYUSH care settings.

### Supplementary Information

**Additional file 1:** **Supplementary File S1.** Multimorbidity Assessment Questionnaire for Primary Care (MAQ-PC). **Table S2.** Prevalence of various chronic conditions across AYUSH facilities. **Supplementary Table S3.** Factors associated with multimorbidity among patients visiting Ayurveda primary care clinics. **Supplementary Table S4.** Factors associated with multimorbidity among patients attending Homoeopathy primary care clinics. **Supplementary Table S5.** Factors associated with multimorbidity among patients attending Unani primary care clinics.

## Data Availability

All data pertaining to the present study may be available after reasonable request to the corresponding authors.

## Abbreviations

AYUSH: Ayurveda, Yoga and Naturopathy, Unani, Siddha, Sowa Rigpa and Homeopathy
LMICs: Low-and middle-income countries
CI: Confidence Interval
AOR: Adjusted Odds Ratio
MAQ-PC: Multimorbidity Assessment Questionnaire for Primary Care

## References

[1] MacMahon S (2018). Multimorbidity: A Priority for Global Health Research.

[2] Asogwa OA, Boateng D, Marzà-Florensa A, Peters S, Levitt N, van Olmen J, Klipstein-Grobusch K (2022). Multimorbidity of non-communicable diseases in low-income and middle-income countries: a systematic review and meta-analysis. BMJ Open.

[3] Arokiasamy P (2018). India's escalating burden of non-communicable diseases. Lancet Glob Health.

[4] Skou ST, Mair FS, Fortin M, Guthrie B, Nunes BP, Miranda JJ, Boyd CM, Pati S, Mtenga S, Smith SM (2022). Multimorbidity. Nat Rev Dis Primers.

[5] Sinha A, Kerketta S, Ghosal S, Kanungo S, Lee JT, Pati S (2022). Multimorbidity and Complex Multimorbidity in India: Findings from the 2017–2018 Longitudinal Ageing Study in India (LASI). Int J Environ Res Public Health.

[6] Sinha A, Varanasi R, Pati S (2021). Kaleidoscopic use of World Health Organization's Study on global AGEing and adult health data set to explore multimorbidity and its outcomes in low and middle-income countries: an insider view. J Fam Med Prim Care.

[7] Barik M, Panda SN, Tripathy SS, Sinha A, Ghosal S, Acharya AS, Kanungo S, Pati S (2022). Is multimorbidity associated with higher risk of falls among older adults in India?. BMC Geriatr.

[8] Nwadiugwu MC (2021). Multi-morbidity in the older person: an examination of polypharmacy and socioeconomic status. Front Public Health.

[9] Menotti A, Mulder I, Nissinen A, Giampaoli S, Feskens EJ, Kromhout D (2001). Prevalence of morbidity and multimorbidity in elderly male populations and their impact on 10-year all-cause mortality: The FINE study (Finland, Italy, Netherlands, Elderly). J Clin Epidemiol.

[10] Ryan BL, Bray Jenkyn K, Shariff SZ, Allen B, Glazier RH, Zwarenstein M, Fortin M, Stewart M (2018). Beyond the grey tsunami: a cross-sectional population-based study of multimorbidity in Ontario. Can J Public Health.

[11] Puri P, Sinha A, Mahapatra P, Pati S (2022). Multimorbidity among midlife women in India: well-being beyond reproductive age. BMC Womens Health.

[12] Kalra S, Baruah MP, Unnikrishnan AG (2017). Responsible patient-centered care. Indian J Endocrinol Metab.

[13] Violan C, Foguet-Boreu Q, Flores-Mateo G, Salisbury C, Blom J, Freitag M, Glynn L, Muth C, Valderas JM (2014). Prevalence, determinants and patterns of multimorbidity in primary care: a systematic review of observational studies. PLoS One.

[14] Rudra S, Kalra A, Kumar A, Joe W (2017). Utilization of alternative systems of medicine as health care services in India: evidence on AYUSH care from NSS 2014. PLoS One.

[15] Pengpid S, Peltzer K (2021). Utilization of complementary and traditional medicine practitioners among middle-aged and older adults in India: results of a national survey in 2017–2018. BMC Complement Med Ther.

[16] Samal J, Dehury RK (2018). Utilization, preference, perception and characteristics of people adopting traditional and AYUSH systems of medicine in India: a systematic review. J Complement Integr Med..

[17] Gautam G, Parveen B, Khan MU, Sharma I, Sharma AK, Parveen R, Ahmad S (2021). A systematic review on nephron protective AYUSH drugs as constituents of NEERI-KFT (A traditional Indian polyherbal formulation) for the management of chronic kidney disease. Saudi J Biol Sci.

[18] Thompson E, Viksveen P, Barron S (2016). A patient reported outcome measure in homeopathic clinical practice for long-term conditions. Homeopathy..

[19] Frei H (2015). Homeopathic treatment of multimorbid patients: a prospective outcome study with polarity analysis. Homeopathy.

[20] Pati S, Swain S, Knottnerus JA, Metsemakers JF, van den Akker M (2019). Health related quality of life in multimorbidity: a primary-care based study from Odisha, India. Health Qual Life Outcomes.

[21] Pati S, Swain S, Metsemakers J, Knottnerus JA, van den Akker M (2017). Pattern and severity of multimorbidity among patients attending primary care settings in Odisha, India. PLoS One.

[22] Vargese SS, Mathew E, Johny V, Kurian N, Raju AS (2020). Prevalence and pattern of multimorbidity among adults in a primary care rural setting. Clin Epidemiol Global Health.

[23] Pati S, Hussain MA, Swain S, Salisbury C, Metsemakers JF, Knottnerus JA, Akker MV. Development and validation of a questionnaire to assess multimorbidity in primary care: an Indian experience. BioMed Res Int. 2016;2016. 10.1155/2016/6582487 PMC4761379 26966687

[24] Saleem SM, Jan SS (2021). Modified Kuppuswamy socioeconomic scale updated for the year 2021. Indian J Forensic Community Med.

[25] Kant S, Lohiya A, Ahamed F, Abdulkader RS, Singh AK, Silan V (2018). Comparative morbidity profile of patients attending an Ayurveda clinic and a modern medicine clinic of a primary health center in rural Haryana, India. J Fam Med Prim Care.

[26] Fisher P (2016). Homeopathy and public health: multimorbidity, polypharmacy, antimicrobial resistance, adverse drug reactions and homeopathy [abstract]. Homeopathy.

[27] Carrier H, Zaytseva A, Bocquier A, Villani P, Verdoux H, Fortin M, Verger P (2019). GPs' management of polypharmacy and therapeutic dilemma in patients with multimorbidity: a cross-sectional survey of GPs in France. Br J Gen Pract.

[28] Pati S, Swain S, Hussain MA, Kadam S, Salisbury C (2015). Prevalence, correlates, and outcomes of multimorbidity among patients attending primary care in Odisha India. Ann Fam Med.

[29] Mbizo J, Okafor A, Sutton MA, Leyva B, Stone LM, Olaku O (2018). Complementary and alternative medicine use among persons with multiple chronic conditions: results from the 2012 National Health Interview Survey. BMC Complement Altern Med.

[30] Marengoni A, Angleman S, Melis R, Mangialasche F, Karp A, Garmen A, Meinow B, Fratiglioni L (2011). Aging with multimorbidity: a systematic review of the literature. Ageing Res Rev.

[31] Sinha A, Kerketta S, Ghosal S, Kanungo S, Pati S (2022). Multimorbidity Among Urban Poor in India: Findings From LASI, Wave-1. Front Public Health.

[32] Pati S, Sinha R, Panda M, Puri P, Pati S (2021). Profile of multimorbidity in outpatients attending public healthcare settings: a descriptive cross-sectional study from Odisha, India. J Fam Med Prim Care.

[33] Prenissl J, De Neve JW, Sudharsanan N, Manne-Goehler J, Mohan V, Awasthi A, Prabhakaran D, Roy A, Tandon N, Davies JI, Atun R (2022). Patterns of multimorbidity in India: a nationally representative cross-sectional study of individuals aged 15 to 49 years. PLOS Global Public Health.

[34] Gupta P, Patel SA, Sharma H, Jarhyan P, Sharma R, Prabhakaran D, Tandon N, Mohan S (2022). Burden, patterns, and impact of multimorbidity in North India: findings from a rural population-based study. BMC Public Health.

[35] Kanungo S, Ghosal S, Kerketta S, Sinha A, Mercer SW, Lee JT, Pati S (2021). Association of oral health with multimorbidity among older adults: Findings from the longitudinal ageing study in India, wave-1, 2017–2019. Int J Environ Res Public Health.

[36] Pati S, Sinha A, Ghosal S, Kerketta S, Lee JT, Kanungo S (2022). Family-level multimorbidity among older adults in india: looking through a syndemic lens. Int J Environ Res Public Health.

