# Supplementary material for: Prevalence and correlates of multimorbidity among patients attending AYUSH primary care settings in Delhi-National Capital Region, India
Source: BMC Complement Med Ther. 2023 Nov 29;23:429. doi: 10.1186/s12906-023-04158-7 (PMC10685658; doi:10.1186/s12906-023-04158-7)
Supplement: Supplementary file 1 — Additional file 1: Supplementary File S1. Multimorbidity Assessment Questionnaire for Primary Care (MAQ-PC). Table S2. Prevalence of various chronic conditions across AYUSH facilities. Supplementary Table S3. Factors associated with multimorbidity among patients visiting Ayurveda primary care clinics. Supplementary Table S4. Factors associated with multimorbidity among patients attending Homoeopathy primary care clinics. Supplementary Table S5. Factors associated with multimorbidity among patients attending Unani primary care clinics. [file 12906_2023_4158_MOESM1_ESM.docx]

**Supplementary File S1 :**

**Multimorbidity Assessment Questionnaire for Primary Care (MAQ-PC)**

**Part I Background Information**

1. Date: _____DD_____MM__________YYYY
2. Dispensary name: ……………………………
3. Dispensary Location____________________
4. Name of AYUSH system

0 Ayurveda , 1 Unani  2 Homoeopathy

1. Name of Patient________________________
2. Mobile No.______________________
3. OPD reg. no._______________
4. Age more than 18 years and above

0 No , 1 Yes

1. Informed consent taken to participate in the study

0 No , ,  1 Yes

1. Upload signed consent Form
2. Visiting the dispensary since…………
3. Age in years: ……………………
4. Are you born and brought up in Delhi  yes  No, If no, which state you belong to _________ and since how long you are in Delhi________________ (in years)
5. Sex:

[_]0 Female [_]1 Male [_]2 Others

1. Religion:

[_]0 Hindu [_]1 Muslim [_]2 Christian [_]3 Sikh [ ]4 Others [ ]5 Prefer not to say

1. Marital Status:

[_]0 Never Married [_]1 Currently married

[_]2 Separated/Divorce/ Widow(er) [_]3 Live-in

1. Ethnicity:

[_]0 Scheduled Caste [_]1 Scheduled Tribe

[_]2 OBC [_]3 General

[ ] 4 Prefer not to declare

1. Education:

[_]0 Illiterate [_]1 Primary level

[_]2 High school or Secondary level [_]3 Graduation/Post graduation

[ ] Profession and Honours

1. Occupation:

[_]0 Professional [_]1 Semi Professional

[ ] Clerical/Shop owner/Farmer [ ] Skilled worker

[ ] Semi Skilled worker

[_]2 Unskilled [_]3 Unemployed

1. Family income per month

[_]≥123322 [_]61663-123321

[ ] 46129-61662 [ ] 30831-46128

[ ]18497-30830

[_]6175-18496 [_]≤6175

1. Total monthly Household expenditure………………………..INR
2. Total Health care expenditure per month
3. APL/BPL (as per ration card):

[_]0 APL [_]1 BPL

1. Where have you spent major part of your life?

[_]0 Urban [_]1 Rural

1. Height in _______mts. Weight in ___________ Kg
2. Are you covered under any health insurance?

[_]0 No [_]1 Yes [_]2 Don’t know

If Yes, what kind of insurance

Govt. funded

- 1. RBSY ((Rashtriya Swasthya Bima Yojana)
  2. Govt. funded Ayushman Barat PM-JAY
  3. Govt. funded CGHS
  4. Govt. funded ESIC
  5. Govt funded DGHS
  6. Other Govt. funded, Specify_____________
  7. Employer Supported (other than Govt.) 1 yes 2 No
  8. Arranged by households with insurance companies 1 yes 2 No

1. In the **past 12 months** have you been **admitted** to hospital excluding trauma/accident/Pregnancy/Acute Emergency/Surgery?

[_]0 No [_]1 Yes [_]2 Don’t Remember

1. If yes, number of times you were admitted to the hospital ____________ (Count)
2. If yes, how many nights altogether have you stayed in the hospital? ___________ (Count)
3. If no, number of times you visited to the hospital /primary care____________ (Count)
4. On an average how many days you took leave from work because of chronic illness per month______(count)
5. On an average, how much do you spend on health care per month?......................INR
6. On an average how much do you spend on transportation to visit hospital per month ____________INR
7. On an average how much do you spend towards doctor consultation (per month) _______________ (INR.)
8. On an average how much do you spend towards lab test (past 6 months)_______________INR
9. On an average how much do you spend towards Medication (per months)_______________INR
10. On an average how much do you spend on other supplements (protein powder, herbal products etc.) (per month)__________INR.

**PART II CHRONIC CONDITIONS MORBIDITY PROFILE AND SEVERITY**

**In the past 12 months, have you ever been told by a health professional/doctor that you have any of the following disease/condition?**

| 1. Arthritis | 7. Heart Disease | 13. Cancer | 19. Prostatic Condition | 25 Chronic headache | 31 fungal infection |
| --- | --- | --- | --- | --- | --- |
| 2. Diabetes | 8. Stroke | 14. Chronic Kidney Diseases | 20. Sleep Disorder | - - - 1. Depression | 32 Uterine fibroid/tumor |
| 3. Hypertension | 9. Visual Difficulty | 15. Seizure disorder | 21. Chronic Liver Disorders | 27. Vitiligo | 33 vaginal discharge/ leucorrhoea/ cervicitis |
| 4. Chronic Lung Diseases | 10. Hearing difficulty | 16. Thyroid Disease | 22. Psoriasis | 28. Anxiety disorder | 34. None |
| 5. Acid Peptic Disease | 11. Dementia | 17. Irritable Bowel Syndrome | 23. Eczema | 29. Chronic cervical/neck pain |  |
| 6. Chronic Backache | 12. Alcohol Disorder | 18. Chronic Constipation | 24. Chronic rhinosinusitis | 30 PCOS/PCOD |  |
| Other | other | other | other | other | Other |
|  |  |  |  |  |  |

1. **Instruction: Please ask about each disease suffering from, Tick the diseases the patient is suffering from and then probe further for specific diseases**

| **Disease/ CONDITION CODE** | **Duration of Illness in years** | **Type of health care provider** | **TYPE OF THERAPY (can tick more than one) . Mention the number of medicines and type of medicine (generic only) being taken for this condition and cost per month** | **How much this problem is limiting you in your daily activities?** |
| --- | --- | --- | --- | --- |
| Verified by (tick as appropriate)  [_]medical records  [_]prescriptions |  | [_]0Public/Govt.  [_]1 Private  [_]2 Both | [_] 1 Allopathy,  Number of medication(s) taken____  Type/Name of medication taken_______________  Total cost per month in INR.____ | [ ] 1. Not at all  [ ] 2. A little  [ ] 3. Somewhat  [ ] 4. Quite a bit  [ ] 5. A lot |
|  |  |  | [_] 2 Ayurveda, Number______  Number of medication(s) taken____  Type/Name of medication taken_______________  Total cost per month in INR.____ |  |
|  |  |  | [_] 3 Unani, Number________  Number of medication(s) taken____  Type/Name of medication taken_______________  Total cost per month in INR.____  __ |  |
|  |  |  | [_] 4 Homoeopathy, Number______  Number of medication(s) taken____  Type/Name of medication taken_______________  Total cost per month in INR.____ |  |
|  |  |  | [_] other measures (supplements) if used, mention__________  Number of medication(s) taken____  Type/Name of medication taken_______________  Total cost per month in INR.____ |  |

| **Disease/ CONDITION CODE** | **Duration of Illness in years** | **Type of health care provider** | **TYPE OF THERAPY (can tick more than one) . Mention the number of medicines and type of medicine (generic only) being taken for this condition and cost per month** | **How much this problem is limiting you in your daily activities?** |
| --- | --- | --- | --- | --- |
| Verified by (tick as appropriate)  [_]medical records  [_]prescriptions |  | [_]0Public/Govt.  [_]1 Private  [_]2 Both | [_] 1 Allopathy,  Number of medication(s) taken____  Type/Name of medication taken_______________  Total cost per month in INR.____ | [ ] 1. Not at all  [ ] 2. A little  [ ] 3. Somewhat  [ ] 4. Quite a bit  [ ] 5. A lot |
|  |  |  | [_] 2 Ayurveda, Number______  Number of medication(s) taken____  Type/Name of medication taken_______________  Total cost per month in INR.____ |  |
|  |  |  | [_] 3 Unani, Number________  Number of medication(s) taken____  Type/Name of medication taken_______________  Total cost per month in INR.____  __ |  |
|  |  |  | [_] 4 Homoeopathy, Number______  Number of medication(s) taken____  Type/Name of medication taken_______________  Total cost per month in INR.____ |  |
|  |  |  | [_] other measures (supplements) if used, mention__________  Number of medication(s) taken____  Type/Name of medication taken_______________  Total cost per month in INR.____ |  |
| **Disease/ CONDITION CODE** | **Duration of Illness in years** | **Type of health care provider** | **TYPE OF THERAPY (can tick more than one) . Mention the number of medicines and type of medicine (generic only) being taken for this condition and cost per month** | **How much this problem is limiting you in your daily activities?** |
| Verified by (tick as appropriate)  [_]medical records  [_]prescriptions |  | [_]0Public/Govt.  [_]1 Private  [_]2 Both | [_] 1 Allopathy,  Number of medication(s) taken____  Type/Nmae of medication taken_______________  Total cost per month in INR.____ | [ ] 1. Not at all  [ ] 2. A little  [ ] 3. Somewhat  [ ] 4. Quite a bit  [ ] 5. A lot |
|  |  |  | [_] 2 Ayurveda, Number______  Number of medication(s) taken____  Type/Name of medication taken_______________  Total cost per month in INR.____ |  |
|  |  |  | [_] 3 Unani, Number________  Number of medication(s) taken____  Type/Name of medication taken_______________  Total cost per month in INR.____  __ |  |
|  |  |  | [_] 4 Homoeopathy, Number______  Number of medication(s) taken____  Type/Name of medication taken_______________  Total cost per month in INR.____ |  |
|  |  |  | [_] other measures (supplements) if used, mention__________  Number of medication(s) taken____  Type/Name of medication taken_______________  Total cost per month in INR.____ |  |
| **Disease/ CONDITION CODE** | **Duration of Illness in years** | **Type of health care provider** | **TYPE OF THERAPY (can tick more than one) . Mention the number of medicines and type of medicine (generic only) being taken for this condition and cost per month** | **How much this problem is limiting you in your daily activities?** |
| Verified by (tick as appropriate)  [_]medical records  [_]prescriptions |  | [_]0Public/Govt.  [_]1 Private  [_]2 Both | [_] 1 Allopathy,  Number of medication(s) taken____  Type/Name of medication taken_______________  Total cost per month in INR.____ | [ ] 1. Not at all  [ ] 2. A little  [ ] 3. Somewhat  [ ] 4. Quite a bit  [ ] 5. A lot |
|  |  |  | [_] 2 Ayurveda, Number______  Number of medication(s) taken____  Type/Name of medication taken_______________  Total cost per month in INR.____ |  |
|  |  |  | [_] 3 Unani, Number________  Number of medication(s) taken____  Type/Name of medication taken_______________  Total cost per month in INR.____  __ |  |
|  |  |  | [_] 4 Homoeopathy, Number______  Number of medication(s) taken____  Type/Name of medication taken_______________  Total cost per month in INR.____ |  |
|  |  |  | [_] other measures (supplements) if used, mention__________  Number of medication(s) taken____  Type/Name of medication taken_______________  Total cost per month in INR.____ |  |
| **Disease/ CONDITION CODE** | **Duration of Illness in years** | **Type of health care provider** | **TYPE OF THERAPY (can tick more than one) . Mention the number of medicines and type of medicine (generic only) being taken for this condition and cost per month** | **How much this problem is limiting you in your daily activities?** |
| Verified by (tick as appropriate)  [_]medical records  [_]prescriptions |  | [_]0Public/Govt.  [_]1 Private  [_]2 Both | [_] 1 Allopathy,  Number of medication(s) taken____  Type/Name of medication taken_______________  Total cost per month in INR.____ | [ ] 1. Not at all  [ ] 2. A little  [ ] 3. Somewhat  [ ] 4. Quite a bit  [ ] 5. A lot |
|  |  |  | [_] 2 Ayurveda, Number______  Number of medication(s) taken____  Type/Name of medication taken_______________  Total cost per month in INR.____ |  |
|  |  |  | [_] 3 Unani, Number________  Number of medication(s) taken____  Type/Name of medication taken_______________  Total cost per month in INR.____  __ |  |
|  |  |  | [_] 4 Homoeopathy, Number______  Number of medication(s) taken____  Type/Name of medication taken_______________  Total cost per month in INR.____ |  |
|  |  |  | [_] other measures (supplements) if used, mention__________  Number of medication(s) taken____  Type/Name of medication taken_______________  Total cost per month in INR.____ |  |
| **Disease/ CONDITION CODE** | **Duration of Illness in years** | **Type of health care provider** | **TYPE OF THERAPY (can tick more than one) . Mention the number of medicines and type of medicine (generic only) being taken for this condition and cost per month** | **How much this problem is limiting you in your daily activities?** |
| Verified by (tick as appropriate)  [_]medical records  [_]prescriptions |  | [_]0Public/Govt.  [_]1 Private  [_]2 Both | [_] 1 Allopathy,  Number of medication(s) taken____  Type/Name of medication taken_______________  Total cost per month in INR.____ | [ ] 1. Not at all  [ ] 2. A little  [ ] 3. Somewhat  [ ] 4. Quite a bit  [ ] 5. A lot |
|  |  |  | [_] 2 Ayurveda, Number______  Number of medication(s) taken____  Type/Name of medication taken_______________  Total cost per month in INR.____ |  |
|  |  |  | [_] 3 Unani, Number________  Number of medication(s) taken____  Type/Name of medication taken_______________  Total cost per month in INR.____  __ |  |
|  |  |  | [_] 4 Homoeopathy, Number______  Number of medication(s) taken____  Type/Name of medication taken_______________  Total cost per month in INR.____ |  |
|  |  |  | [_] other measures (supplements) if used, mention__________  Number of medication(s) taken____  Type/Name of medication taken_______________  Total cost per month in INR.____ |  |

**Part III Quality of life and self-rated health**

1. **Quality of Life (EQ-5D-5L)**

| Under each heading, please tick the box that best describes your health TODAY | |
| --- | --- |
| **MOBILITY**   1. I have no problems in walking about 2. I have slight problems in walking about 3. I have moderate problems in walking about 4. I have severe problems in walking about 5. I am unable to walk about | **SELF-CARE**   1. I have no problems washing or dressing myself 2. I have slight problems washing or dressing myself 3. I have moderate problems washing or dressing myself 4. I have severe problems washing or dressing myself 5. I am unable to wash or dress myself |
| **PAIN / DISCOMFORT**   1. I have no pain or discomfort 2. I have slight pain or discomfort 3. I have moderate pain or discomfort 4. I have severe pain or discomfort 5. I have extreme pain or discomfort | **UNUSUAL ACTIVITIES (work, study, housework, family or leisure activities)**   1. I have no problems doing my usual activities. 2. I have slight problems doing my usual activities. 3. I have moderate problems doing my usual activities. 4. I have severe problems doing my usual activities. 5. I am unable to do my usual activities |
| **ANXIETY / DEPRESSION**   1. I am not anxious or depressed 2. I am slightly anxious or depressed 3. I am moderately anxious or depressed 4. I am severely anxious or depressed 5. I am extremely anxious or depressed |  |

**SELFRATED HEALTH**

1. How would you rate your overall physical health? 0 very poor 3 very good health

[_]0 [_]1 [_]2 [_]3

1. How would you rate your overall mental health? 0 very poor 3 very good health

[_]0 [_]1 [_]2 [_]3

1. How would you rate your overall social health? 0 very poor 3 very good health

[_]0 [_]1 [_]2 [_]3

1. **The Multimorbidity Illness Perceptions Scale (MULTIPleS)**

**Ask this question to patients who have more than one disease present else skip this part.**

| These questions are about the thoughts and feelings that people with multiple long-term conditions sometimes experience. Please indicate how much you agree with each statement by circling one number.  Strongly Disagree Strongly Agree | | | | | | | | |  |
| --- | --- | --- | --- | --- | --- | --- | --- | --- | --- |
| 1 | One of my conditions is more serious than the others. | 0 | 1 | 2 | 3 | 4 | 5 | |  |
| 2 | Time spent managing my conditions has made it more difficult to carry out my usual activities. | 0 | 1 | 2 | 3 | 4 | 5 | |  |
| 3 | I feel so overwhelmed by the treatment for one condition it is hard to manage any others. | 0 | 1 | 2 | 3 | 4 | 5 | |  |
| 4 | The causes of my conditions are linked. | 0 | 1 | 2 | 3 | 4 | 5 | |  |
| 5 | It is difficult to take all my medications the way I am supposed to. | 0 | 1 | 2 | 3 | 4 | 5 | |  |
| 6 | Time spent managing my condition has limited my activities. | 0 | 1 | 2 | 3 | 4 | 5 | |  |
| 7 | One of my conditions is more worrying than the others. | 0 | 1 | 2 | 3 | 4 | 5 | |  |
| 8 | Taking different medications for each of my conditions has caused me problems. | 0 | 1 | 2 | 3 | 4 | 5 | |  |
| 9 | I don't like mixing medications for different conditions. | 0 | 1 | 2 | 3 | 4 | 5 | |  |
| 10 | Having more than one condition makes my treatments less effective. | 0 | 1 | 2 | 3 | 4 | 5 | |  |
| 11 | One of my conditions has caused another. | 0 | 1 | 2 | 3 | 4 | 5 | |  |
| 12 | One of my conditions dominates the others. | 0 | 1 | 2 | 3 | 4 | 5 | |  |
| 13 | My conditions interact with each other. | 0 | 1 | 2 | 3 | 4 | 5 | |  |
| 14 | Having more than one condition makes it difficult to get the best available treatment. | 0 | 1 | 2 | 3 | 4 | 5 | |  |
| 15 | Time spent managing my conditions has reduced my social life. | 0 | 1 | 2 | 3 | 4 | 5 | |  |
| 16 | One of my conditions has more of an impact on my life. | 0 | 1 | 2 | 3 | 4 | 5 | |  |
| 17 | Having more than one condition makes me unhappy. | 0 | 1 | 2 | 3 | 4 | | 5 | |
| 18 | Having more than one condition makes me more anxious. | 0 | 1 | 2 | 3 | 4 | | 5 | |
| 19 | Having more than one condition makes me angry or frustrated. | 0 | 1 | 2 | 3 | 4 | | 5 | |
| 20 | Having more than one health problem makes me feel sad. | 0 | 1 | 2 | 3 | 4 | | 5 | |
| 21 | Having more than one condition makes me more irritable. | 0 | 1 | 2 | 3 | 4 | | 5 | |
| 22 | If I feel sad or depressed, managing my conditions is a struggle. | 0 | 1 | 2 | 3 | 4 | | 5 | |

**Thank you for your participation.**

| Name of the Investigating doctor | Signature of the Investigating doctor |
| --- | --- |

**Table S2: Prevalence of various chronic conditions across AYUSH facilities**

| **Disease condition** | **Total (N=943)** | **Ayurveda (N=246)** | **Unani (N=121)** | **Homoeopathy (N=576)** | **P-value** |
| --- | --- | --- | --- | --- | --- |
|  | n (%) | n (%) | n (%) | n (%) |  |
| Diabetes | 139(14.7) | 51(20.7) | 22(18.2) | 66(11.5) | **0.001** |
| Hypertension | 135(14.3) | 39(15.9) | 23(19) | 73(12.7) | 0.141 |
| Heart disease | 24(2.5) | 7(2.8) | 5(4.1) | 12(2.1) | 0.404 |
| Chronic lung disease (including asthma, allergic bronchitis) | 40(4.3) | 4(1.6) | 9(7.4) | 27 (4.7) | **0.024** |
| Acid peptic disease | 88(9.3) | 25(10.2) | 22(18.2) | 41(7.1) | **0.001** |
| Skin diseases (psoriasis, vitiligo, dermatitis, eczema, melasma, acne, fungal infection, corn, warts) | 123(13.1) | 26 (10.6) | 8 (6.6) | 90(25.6) | **0.015** |
| Musculoskeletal diseases (arthritis, gout, sciatica, neck pain and low back pain, frozen shoulder) | 213 | 64(26.0) | 32 (26.4) | 117(20.3) | 0.111 |
| Cardio-metabolic diseases | 243(25.8) | 81(32.9) | 36(29.8) | 126(21.9) | **0.002** |
| Visual difficulty | 23(2.4) | 9(3.7) | 1(0.8) | 13(2.3) | 0.230 |
| Hearing difficulty | 3(0.3) | 1(0.4) | 0(0) | 2(0.3) | 0.794 |
| Dementia | 3 (0.3) | 1(0.4) | 1(0.8) | 1 (0.2) | 0.490 |
| Alcohol disorder | 4(0.4) | 2(0.8) | 2(1.7) | 0(0) | **0.022** |
| Cancer | 2(0.2) | 0 | 0 | 2(0.3) | 0.528 |
| Chronic kidney disease | 13(1.4) | 5(2) | 1(0.8) | 7(1.2) | 0.560 |
| Seizure | 5(0.5) | 1(0.4) | 2(1.7) | 2(0.3) | 0.189 |
| Thyroid disorder | 94(10) | 23(9.3) | 14(11.6) | 57(9.9) | 0.797 |
| Irritable bowel syndrome | 16(1.7) | 5(2) | 1(0.8) | 10(1.7) | 0.697 |
| Chronic constipation | 74(7.8) | 34(13.8) | 10(8.3) | 30(5.2) | **0.000142** |
| Prostate disorder | 19(2.0) | 5(2) | 2(1.7) | 12(2.1) | 0.954 |
| Sleep disorder | 19(2) | 6(2.4) | 0(0) | 13(2.3) | 0.237 |
| Chronic liver disorder | 30(3.2) | 8(3.3) | 2(1.7) | 20(3.5) | 0.583 |
| Chronic sinusitis | 65(6.9) | 10(4.1) | 10(8.3) | 45(7.8) | 0.124 |
| Chronic headache | 35(3.7) | 6(2.4) | 5(4.1) | 24(4.2) | 0.470 |
| Mental disease (Depression/Anxiety) | 28(3.0) | 9 (3.7) | 2(1.7) | 17(3) | 0.567 |
| Renal calculi | 16(1.7) | 2 (0.8) | 3(2.5) | 11(1.9) | 0.416 |
| Cholelithiasis | 5(0.5) | 0(0) | 1 (0.8) | 4(0.7) | 0.405 |
| Piles | 44(4.7) | 14(5.7) | 8(6.6) | 22(3.8) | 0.281 |
| Alopecia | 11(1.2) | 0(0) | 2(1.7) | 9(1.6) | 0.140 |
| Leucorrhoea | 44(4.7) | 12 (4.9) | 3(2.5) | 29(5.0) | 0.472 |
| Uterine fibroid | 20(2.1) | 4(1.8) | 1(0.6) | 15(2.6) | 0.384 |
| PCOD (Poly Cystic Ovarian Disease) | 19(2.0) | 6(2.4) | 2(1.7) | 11(1.9) | 0.845 |
| Chronic headache | 35(3.7) | 6(2.4) | 5(4.1) | 24(4.2) | 0.470 |

**Supplementary Table S3: Factors associated with multimorbidity among patients visiting Ayurveda primary care clinics**

| **Variable** | **Total (n=246)** | **No/ Single morbidity (n=140)** | **Multimorbidity (n=106)** | **OR** | **p-value** | **AOR** | **p-value** |
| --- | --- | --- | --- | --- | --- | --- | --- |
| **Age (Years)** |  |  |  |  |  |  |  |
| 18-29 | 39(15.9) | 29(74.4) | 10(25.6) | Ref. |  | Ref. |  |
| 30-39 | 51(20.7) | 29(20.7) | 22(20.8) | 2.20(0.88 to 5.45) | 0.089 | 3.40(1.06 to 10.92) | **0.040** |
| 40-49 | 77(31.3) | 38(48.4) | 39(50.6) | 2.97(1.27 to 6.93) | 0.012 | 5.68(1.19 to 16.93) | **0.002** |
| 50-59 | 46(18.7) | 27(58.7) | 19(41.3) | 2.04(0.80 to 5.16) | 0.132 | 5.20(1.53 to 17.66) | **0.008** |
| 60-69 | 24(9.8) | 11(45.8) | 13(54.2) | 3.42(1.16 to 10.06) | 0.025 | 4.40(0.99 to 19.44) | 0.051 |
| ≥70 | 9(3.7) | 6(66.7) | 3(33.3) | 1.45(0.30 to 6.90) | 0.641 | 2.67(0.34 to 20.83) | 0.490 |
| **Sex** |  |  |  |  |  |  |  |
| Male | 139(43.5) | 82(59.0) | 57(41.0) | Ref. |  | Ref. |  |
| Female | 107 (56.5) | 58(54.2) | 49(45.8) | 1.21(0.73 to 2.02) | 0.452 | 1.37(0.56 to 3.34) | 0.490 |
| **Education** |  |  |  |  |  |  |  |
| No formal education | 14(5.7) | 4(28.6) | 10(71.4) | Ref. |  | Ref**.** |  |
| Primary | 53(21.5) | 24(45.3) | 29(54.7) | 0.48(0.13 to 1.73) | 0.265 | 0.55(0.11 to 2.57) | 0.451 |
| Secondary and above | 179(72.8) | 112(62.6) | 67(37.4) | 0.23(0.07 to 0.79) | 0.019 | 0.33(0.75 to 1.46) | 0.144 |
| **Occupation** |  |  |  |  |  |  |  |
| Unemployed | 93(37.8) | 54(58.1) | 39(41.9) | Ref. |  | Ref. |  |
| employed | 152(62.2) | 86(56.2) | 67(43.8) | 1.07(0.64 to 1.81) | 0.776 | 1.09(0.41 to 2.91) | 0.859 |
| **Marital status** |  |  |  |  |  |  |  |
| Single | 62(25.2) | 24(38.7) | 38(61.3) | Ref. |  | Ref. |  |
| Married | 184(74.8) | 116(63.0) | 68(37.0) | 0.37(0.20 to 0.66) | 0.001 | 0.25(0.10 to 0.59) | **0.002** |
| **Ethnicity** |  |  |  |  |  |  |  |
| Aboriginal | 31(17.1) | 14(45.2) | 17(54.8) | Ref. |  | Ref**.** |  |
| Non aboriginal | 150(82.9) | 71(47.3) | 79(52.7) | 0.91(0.42 to 1.99) | 0.916 | 0.91(0.38 to 2.19) | 0. 840 |
| **Socio-economic class** |  |  |  |  |  |  |  |
| Lower | 141(57.3) | 81(57.5) | 60(42.6) | Ref. |  | Ref. |  |
| Middle | 96(39.0) | 53(55.2) | 43(44.8) | 0.67(0.16 to 2.80) | 0.589 | 0.83(0.08 to 7.84) | 0.876 |
| Upper | 9(3.7) | 6(66.7) | 3(33.3) | 1.09(0.64 to 1.84) | 0.733 | 2.17(0.94 to 5.03) | 0.069 |

Data are presented as n (%); COR: crude odds ratio; AOR: Adjusted Odds Ratio; 95% CI: 95% Confidence interval

**Supplementary Table S4: Factors associated with multimorbidity among patients attending Homoeopathy primary care clinics**

| **Variable** | **Total (n=943)** | **No/ Single morbidity (n=571)** | **Multimorbidity (n=372)** | **OR** | **p-value** | **AOR** | **p-value** |
| --- | --- | --- | --- | --- | --- | --- | --- |
| **Age (Years)** |  |  |  |  |  |  |  |
| 18-29 | 129(22.4) | 102(79.8) | 20(20.2) | Ref. |  | Ref. |  |
| 30-39 | 149(25.9) | 106(71.1) | 26(20.2) | 1.60(0.92 to 2.80) | 0.095 | 1.66(0.77 to 3.53) | 0.190 |
| 40-49 | 136(23.6) | 72(52.9) | 64(47.1) | 3.52(2.03 to 6.08) | 0.0001 | 3.54(1.64 to 7.62) | **0.001** |
| 50-59 | 91(15.8) | 50(54.9) | 41(45.1) | 3.24(1.78 to 5.89) | 0.0001 | 3.58(1.54 to 8.31) | **0.003** |
| 60-69 | 43(7.5) | 23(53.5) | 20(46.5) | 3.44(1.64 to 7.20) | 0.001 | 4.50(1.62 to 12.46) | **0.004** |
| ≥70 | 28(4.9) | 9(32.1) | 10(67.9) | 8.36(3.39 to 20.61) | 0.0001 | 13.23(3.97 to 44.08) | 0.0001 |
| **Sex** |  |  |  |  |  |  |  |
| Male | 245(42.5) | 162(66.1) | 83(33.9) | Ref. |  | Ref. |  |
| Female | 331(57.5) | 201(60.7) | 130(39.3) | 1.26(0.89 to 1.78) | 0.180 | 1.97(1.13 to 3.44) | **0.016** |
| **Education** |  |  |  |  |  |  |  |
| No formal education | 17(3.0) | 8(47.1) | 9(52.9) | Ref. |  | Ref**.** |  |
| Primary | 110(19.1) | 65(59.1) | 45(40.9) | 0.61(0.22 to 1.71) | 0.353 | 1.02(0.27 to 3.81) | 0.972 |
| Secondary and above | 449(78.0) | 43 (64.6) | 159(35.4) | 0.48(0.18 to 1.28) | 0.147 | 0.79(0.22 to 2.86) | 0.730 |
| **Occupation** |  |  |  |  |  |  |  |
| Unemployed | 264(45.8) | 161(61.0) | 103(39.0) | Ref. |  | Ref. |  |
| employed | 312(54.2) | 202(64.7) | 110(35.3) | 0.85(0.60 to 1.19) | 0.352 | 1.06(0.58 to 1.92) | 0.845 |
| **Marital status** |  |  |  |  |  |  |  |
| Single | 139(24.1) | 101(72.7) | 38(27.3) | Ref. |  | Ref. |  |
| Married | 437(75.9) | 262(60.0) | 175(40.0) | 1.77(1.16 to 2.70) | 0.007 | 1.34 (0.70 to 2.56) | 0.318 |
| **Ethnicity** |  |  |  |  |  |  |  |
| Aboriginal | 91(22.1) | 62(68.1) | 29(31.9) | Ref. |  | Ref**.** |  |
| Non aboriginal | 321(77.9) | 193(60.1) | 128(39.9) | 1.48(0.86 to 2.33) | 0.166 | 1.31(0.77 to 2.23) | 0.376 |
| **Socio-economic class** |  |  |  |  |  |  |  |
| Lower | 297(51.6) | 191(64.3) | 106(35.7) | Ref. |  | Ref. |  |
| Middle | 247(42.9) | 152(61.5) | 95(38.5) | 1.08(0.50 to 2.29) | 0.839 | 1.31(0.48 to 3.56) | 0.592 |
| Upper | 32(5.6) | (62.5) | 12(37.5) | 1.26(0.79 to 1.59) | 0.505 | 1.96(1.11 to 3.46) | **0.020** |

Data are presented in n(%); COR: crude odds ratio; AOR: Absolute Odds Ratio; 95% CI: 95% Confidence interval

**Supplementary Table S5: Factors associated with multimorbidity among patients attending Unani primary care clinics**

| **Variable** | **Total (N=121)** | **No/ Single morbidity (N=68)** | **Multimorbidity (n=53)** | **COR** | **p-value** | **AOR** | **p-value** |
| --- | --- | --- | --- | --- | --- | --- | --- |
| **Age (Years)** |  |  |  |  |  |  |  |
| 18-29 | 24(19.8) | 19(79.2) | 5(20.8) |  |  | Ref. |  |
| 30-39 | 33(27.3) | 23(69.7) | 10(30.3) | 1.65(0.48 to 5.61) | 0.425 | 2.55(0.57 to 11.65) | 0.215 |
| 40-49 | 25(20.7) | 13(52.0) | 12(48.0) | 3.50(0.99 to 12.35) | 0.051 | 3.38(0.67 to 17.01) | 0.139 |
| 50-59 | 20(16.5) | 7(35.0) | 13(65.0) | 7.05(1.83 to 27.14) | 0.004 | 5.17(0.86 to 31.06) | 0.072 |
| 60-69 | 10(8.3) | 4(40) | 6(60) | 5.70(1.14 to 28.33) | 0.033 | 4.04(0.24 to 66.47) | 0.328 |
| ≥70 | 09(7.4) | 2(22.2) | 7(77.8) | 13.30(2.08 to 84.98) | 0.006 | 5.47(0.61 to 49.88) | 0.132 |
| **Sex** |  |  |  |  |  |  |  |
| Male | 61(50.4) | 32(60.0) | 29(40.0) |  |  | Ref. |  |
| Female | 60(49.6) | 68(56.2) | 53(43.8) | 1.35(0.66 to 2.79) | 0.404 | 1.23(0.35 to 4.26) | 0.742 |
| **Education** |  |  |  |  |  |  |  |
| No formal education | 04(3.3) | 2(50) | 2(50) |  |  | Ref. |  |
| Primary | 40(33.1) | 23(57.5) | 17(42.5) | 0.73(0.09 to 5.78) | 0.773 | 0.63 (0.03 to 12.28) | 0.761 |
| Secondary and above | 77(63.6) | 43(55.8) | 34(44.2) | 0.79(0.10 to 5.90) | 0.819 | 0.25(0.01 to 5.75) | 0.392 |
| **Occupation** |  |  |  |  |  |  |  |
| Unemployed | 59(48.8) | 26(38.2) | 33(62.3) | Ref. |  | Ref. |  |
| Employed | 62(51.2) | 42(67.7) | 20(32.3) | 0.37(0.17 to 0.78) | 0.009 | 0.11(0.02 to 0.56) | 0.008 |
| **Marital status** |  |  |  |  |  |  |  |
| Single | 32(26.4) | 22(68.8) | 10(31.3) | Ref. |  | Ref. |  |
| Married | 89(73.6) | 46(51.7) | 43(48.3) | 2.05(0.87 to 4.83) | 0.098 | 1.08(0.29 to 3.99) | 0.901 |
| **Ethnicity** |  |  |  |  |  |  |  |
| Aboriginal | 09(9.5) | 6(66.7) | 3(33.3) | Ref. |  | Ref**.** |  |
| Non aboriginal | 86(90.5) | 56(65.1) | 30(34.9) | 1.07(0.25 to 4.59) | 0.926 | 1.05(0.17 to 6.38) | 0.958 |
| **Socio-economic class** |  |  |  |  |  |  |  |
| Lower | 90(74.4) |  |  | Ref. |  | Ref. |  |
| Middle | 28(23.1) | 55(61.1) | 35(38.9) | 3.14(0.27 to 35.97) | 0.357 | 16.27(0.77 to 340.19) | 0.072 |
| Upper | 3(2.5) | 1(33.3) | 2(66.7) | 2.09(0.88 to 4.95) | 0.092 | 12.(2.09 to 75.94) | **0.006** |

Data are presented in n(%); COR: crude odds ratio; AOR: Absolute Odds Ratio; 95% CI: 95% Confidence interval
